# Potentiated Interaction between Ineffective Doses of Budesonide and Formoterol to Control the Inhaled Cadmium-Induced Up-Regulation of Metalloproteinases and Acute Pulmonary Inflammation in Rats

**DOI:** 10.1371/journal.pone.0109136

**Published:** 2014-10-14

**Authors:** Wenhui Zhang, Jianming Zhi, Yongyao Cui, Fan Zhang, Adélite Habyarimana, Carole Cambier, Pascal Gustin

**Affiliations:** 1 Department of Physiology, Shanghai Jiao Tong University School of Medicine, Shanghai, People's Republic of China; 2 Department of Pharmacology, Shanghai Jiao Tong University School of Medicine, Shanghai, People's Republic of China; 3 Department of Pathology, Shanghai Jiao Tong University School of Medicine, Shanghai, People's Republic of China; 4 Department for Functional Sciences B41, Faculty of Veterinary Medicine, University of Liège, Liège, Belgium; Cincinnati Children's Hospital Medical Center, United States of America

## Abstract

The anti-inflammatory properties of glucocorticoids are well known but their protective effects exerted with a low potency against heavy metals-induced pulmonary inflammation remain unclear. In this study, a model of acute pulmonary inflammation induced by a single inhalation of cadmium in male Sprague-Dawley rats was used to investigate whether formoterol can improve the anti-inflammatory effects of budesonide. The cadmium-related inflammatory responses, including matrix metalloproteinase-9 (MMP-9) activity, were evaluated. Compared to the values obtained in rats exposed to cadmium, pretreatment of inhaled budesonide (0.5 mg/15 ml) elicited a significant decrease in total cell and neutrophil counts in bronchoalveolar lavage fluid (BALF) associated with a significant reduction of MMP-9 activity which was highly correlated with the number of inflammatory cells in BALF. Additionally, cadmium-induced lung injuries characterized by inflammatory cell infiltration within alveoli and the interstitium were attenuated by the pre-treatment of budesonide. Though the low concentration of budesonide (0.25 mg/15 ml) exerted a very limited inhibitory effects in the present rat model, its combination with an inefficient concentration of formoterol (0.5 mg/30 ml) showed an enhanced inhibitory effect on neutrophil and total cell counts as well as on the histological lung injuries associated with a potentiation of inhibition on the MMP-9 activity. In conclusion, high concentration of budesonide alone could partially protect the lungs against cadmium exposure induced-acute neutrophilic pulmonary inflammation via the inhibition of MMP-9 activity. The combination with formoterol could enhance the protective effects of both drugs, suggesting a new therapeutic strategy for the treatment of heavy metals-induced lung diseases.

## Introduction

Cadmium is listed by the Agency for Toxic Substances and Disease Registry as the world's seventh largest hazardous substance. It is also classified as a Group 1 carcinogen by International Agency for Research on Cancer (IARC) [1]. Inhalation is an important route of cadmium exposure for occupationally and non-occupationally exposed population. Indeed, tobacco smoke can be an important vector for cadmium in smokers but also in passive smokers due to the high concentrations that can be reached in indoor air [2]. Cadmium is also widely used in some industries and the majority of cadmium present in atmosphere is the result of fossil fuel combustion and municipal waste incineration [3]. Lungs thus became a toxicological target as illustrated by the marked deficit in lung function correlated with an increase in urinary cadmium concentration which has been found in workers exposed to cadmium in jewelry workshops [4]. The involvement of cadmium in obstructive lung diseases (OLD), including chronic obstructive pulmonary disease (COPD), is also supported by several studies in human, animal models and cell cultures illustrating the deleterious effects of this heavy metal and mechanisms of action on pulmonary tissue [5]–[8]. Acute exposure to cadmium also induces deterioration in lung function and neutrophilic infiltration which is a dominant component of COPD especially during acute exacerbations of this chronic inflammatory process [9].

Experimental models mimicking the pathological characteristics of these inflammatory diseases and allowing pharmacological research about inhaled cadmium-induced acute and chronic pulmonary inflammation have been developed in rats [10]–[12]. As in naturally occurring diseases, matrix metalloproteinases (MMPs) play an important role in rats, especially MMP-9 produced by resident and inflammatory cells, especially macrophages [12]–[15]. Inhibition of these enzymes provides a very effective protective effect against lung injuries especially against cadmium-induced neutrophils migration [11], [16], [17]. Likewise in patients with COPD, this neutrophilic inflammation appears rather poorly sensitive to the action of glucocorticoids (GCs) nevertheless recognized as the most powerful anti-inflammatory agents to treat inflammatory diseases as asthma [18], [19]. The absence of influence of GCs on MMPs activity has been suggested to be partially responsible of this lack of efficacy [20] but their action on pulmonary MMPs activity especially in relation with heavy metals inhalation remains controversial and further studies are necessary to investigate how to improve their anti-inflammatory effects [19], [21], [22].

Recovery of these anti-inflammatory effects is a key therapeutic challenge [23], [24]. The anti-inflammatory effects of β_2_-adrenergic receptor agonists, classically used as bronchodilators, have been recently reviewed and previously demonstrated in rats exposed to cadmium [11], [12], [25]. Clinical benefits provided by the combination of long acting β_2_-adrenergic receptor agonists (LABAs) with GCs have been reported in diseases as asthma and sometimes in COPD [26]–[29] as well as *in vitro* [30]–[32]. However, nothing is known about this interaction and the possible role played by MMPs in refractory models of lung inflammation induced by heavy metals [30], [31], [33].

By using the rat model of acute pulmonary inflammation associated with an elevated MMP-9 activity induced by a single inhalation of cadmium, the aim of this study was to determine whether inefficient concentrations of inhaled budesonide, alone or in combination with formoterol, can exert a protective effect against cadmium-induced acute pulmonary inflammation in rats. We also examined whether the expected anti-inflammatory effects of budesonide and/or formoterol were associated with a modulation of MMP-9 activity.

## Materials and Methods

### Ethics Statement

Male Sprague-Dawley rats (n = 84) weighing 250–300 g from Shanghai SLAC Laboratory Animal Co. Ltd. were used. All experimental procedures were conducted in accordance with the guidelines for the care and use of laboratory animals from the Experimental Animal Care and Use Committee of Shanghai Jiao Tong University. Animals were housed in the animal facility of the laboratory with a 12∶12 h light-dark cycle at a constant ambient temperature (23±2°C) and humidity (60%±5%), with free access to water and food, for at least 24 h before experimentation. All efforts were made to minimize suffering. All experimental protocols were approved by the Experimental Animal Care and Use Committee of Shanghai Jiao Tong University School of Medicine.

Animals were placed in an exposure chamber (length×width×height: 50 cm×50 cm×37 cm) to be exposed to drugs and cadmium by an ultrasonic nebulizer (402AI, YuYue Medical Equipment Co., Ltd. Jiangsu, China) known to generate particles of diameter ranging 1–5 µm. The aerosol output was 3 ml/min which was propelled by the airflow of 30 L/min into the chamber. The animals were allowed to move freely during exposure. Two lateral opening 10 mm in diameter in the chamber ensured a regular dispersion of the aerosol. The whole system was placed within an extractor hood ensuring a safe aerosol evacuation. The animals were sacrificed 24 h later by a lethal intraperitoneal injection of pentobarbital (200 mg/kg), a time corresponding to the maximum inflammatory response induced by cadmium. The lungs were removed from the chest. As described previously [10], bronchoalveolar lavage fluid (BALF) was collected by flushing 8 ml of saline two times successively in the right lung through a cannula located in the main bronchus. BALF recovery ranged from 80 to 90% and did not differ between groups of animals. The liquid was then collected for subsequent BALF analysis. The left lung tissue was fixed for histological examination.

### Agents and dose selection

Cadmium chloride, formoterol fumarate and budesonide were obtained from Sigma (St Louis, USA). Cadmium chloride was prepared in saline to get a 0.1% solution. Formoterol fumarate and budesonide were first dissolved in DMSO and then in saline to get a 0.1% DMSO in saline solution. Two different concentrations of formoterol (0.5 mg/30 ml, 1 mg/30 ml of nebulized solution) have been selected according to our previous study demonstrating the bronchodilating effects and anti-inflammatory properties of this drug [11]. The aim of the study being to check whether low doses of glucocorticoids can interact with formoterol to better control the cadmium-induced pulmonary inflammation, only two doses were assessed. The doses of budesonide have been determined during preliminary assays demonstrating the anti-inflammatory effects of this agent in rats exposed during 1 h to cadmium. Two doses of budesonide (0.25 mg/15 ml, 0.5 mg/15 ml of nebulized solution) inducing mild to moderate anti-inflammatory effects were selected in the present study.

### Study design and experimental protocols

Animals were pretreated with formoterol, budesonide or a combination of both drugs (0.5 mg/30 ml of formoterol combined with 0.25 mg/15 ml of budesonide) before the cadmium (5 groups, n = 6 per group) or saline (5 groups, n = 6 per group) exposure. Formoterol (30 ml) and budesonide (15 ml) solutions were nebulized at the same rate during 15 and 10 min respectively. When combined, formoterol was first nebulized immediately followed by budesonide. Three corresponding cadmium-exposed control groups were also investigated. Before inhalation of the cadmium solution, animals of these groups were exposed to nebulized solution (0.1% DMSO solution of 0.9% NaCl) during 15 min (n = 6), 10 min (n = 6) or 25 min (n = 6). As the duration of the pretreatment period with the vehicle had no influence on the response to the cadmium, all data from the cadmium-exposed animals were pooled to obtain a single cadmium control group.

Finally a sham group (n = 6) underwent the same protocol for 85 min with the corresponding vehicles (25 min with 0.1% DMSO in 0.9% NaCl solution) followed by a saline inhalation for 60 min.

### Cytological analysis in bronchoalveolar lavage fluid (BALF)

BALF was centrifuged for 15 min at 4°C (450 *g*) and the supernatant was fractioned into 1 ml samples, which were kept at −80°C for further analysis. A total cell count was performed using a Thomas cell. 150 µl of BALF was used for differential cell count by cytospin centrifugation and May Grünwald Giemsa staining.

### Determination of matrix metalloproteinase activity in BALF

The activity of MMP-9 was detected by gelatin zymography as previously described [10]. Briefly, 10 µl of supernatant of BALF was added to Laemmli buffer at a 1∶1 ratio. The samples were subjected to electrophoresis on 10% acrylamide SDS gels containing porcine skin gelatine (1%, Sigma, St. Louis, USA). After loading onto gelatin gels with standards, the gels were then washed twice in Triton X-100 2% for 30 min followed by an incubation at 37°C overnight. After staining with Coomassie blue and destaining, gelatinolytic activity appeared as unstained zones against a blue background. Quantification of MMP activity was analyzed with Image J (Image Processing and Analysis in Java). Results were expressed as average arbitrary units (AU) corresponding to pixel density×mm^2^ for the bands of proteolysis normalized by the same value calculated for a known amounts of standard (human proenzyme MMP-9, San Diego, CA, USA). As the latent and active bands were so close that it was difficult to accurately analyze these two forms of MMPs respectively, the latent and active bands were taken together for analysis. In the sham group, MMP-9 activity was sometimes undetectable. So a 0 value was attributed to the corresponding samples.

### Detection of cytokines and chemokines in BALF

The levels of IL-1β, TNF-α and IL-8 in BALF were analyzed by ELISA according to the manufacturer's instruction (Rapidbio Lab, California, USA).

### Lung histological examination

The left lung was harvested and fixed in 10% phosphate buffered formalin. After 24 h of fixation, the median portion was cut and embedded in paraffin. The paraffin block per lung was cut into 2–3 µm transversal slices and stained with hematoxylin and eosin (H&E). Three nonoverlapping slices from each lung sample were randomly screened. Care was taken to avoid regions containing pleura or large bronchi. Slices were examined by a pathologist blinded to sample groups. The degree of microscopic lung injuries was analyzed using a semi quantitative method. Four fields per slice were randomly selected and evaluated for several variables (neutrophil and macrophage infiltration, edema and hemorrhage) according to severity and extent of inflammatory responses, and relative scores were attributed. The mean of three slices per lung was taken as representative value of each animal. For each slice, the extent of injury was assigned a score of 0 (injury less than 5% of the field), 1 (5–20% of the field), 2 (20%–40%), or 3 (>40%), and the severity was graded as 0 (normal, no cellular infiltration), 1 (mild injury, scarcely neutrophil or macrophage infiltration), 2 (moderate injury, moderate neutrophil or macrophage infiltration with mild edema or hemorrhage), or 3 (severe injury, severe inflammatory infiltration with moderate edema or hemorrhage). The extent and the severity score were expressed respectively by mean ± SD of every group.

### Statistical analysis of results

All data were normally distributed and presented as means ± S.D., and were analyzed by one-way analysis of variance (ANOVA) by using graphpad prism 5.0 (Graphpad Software. San Diego, CA). Student-Newman-Keuls tests were performed subsequently for comparison between groups. The correlation analysis was performed by linear regression and the value r was measured as coefficient of correlation. P<0.05 was considered as statistically significant.

## Results

### Effects of formoterol and/or budesonide on differential BALF cell counts

**Figure 1:**
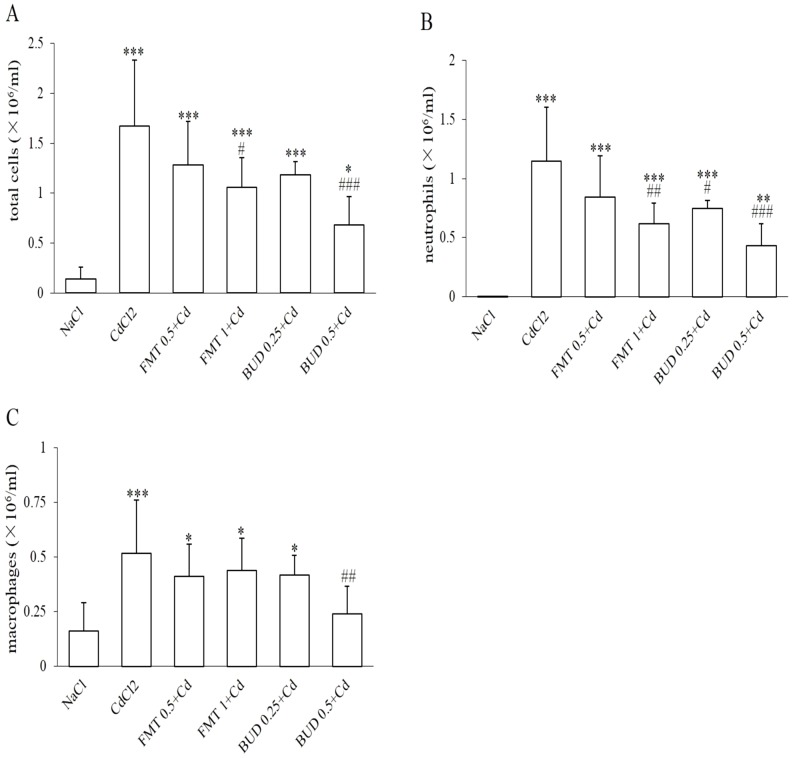
Protective effects of formoterol and budesonide on the concentrations of total cells (A), neutrophils (B) and macrophages (C) in bronchoalveolar lavage fluid in rats exposed to cadmium. CdCl_2_: 0.1% CdCl_2_ cadmium group; FMT 0.5+Cd and FMT 1+Cd: animals pretreated with increasing concentrations of formoterol (0.5 mg/30 ml; 1 mg/30 ml respectively) before cadmium inhalation; BUD 0.25+Cd and BUD 0.5+Cd: animals exposed to budesonide (0.25 mg/15 ml and 0.5 mg/15 ml respectively) followed by cadmium exposure; * Indicates a significant difference in comparison with sham group (* P<0.05, ** P<0.01, *** P<0.001); # indicates a significant difference in comparison with cadmium-exposed group (# P<0.05, ## P<0.01, ###P<0.001).

A single cadmium exposure induced a significant increase in total cell number in BALF which was attributed to a marked increase in neutrophil and macrophage concentrations in BALF (Fig. 1A–C). Pretreatment of healthy rats with formoterol and/or budesonide had no effect on BALF cell counts as compared to sham group (data not shown).

Though the low concentration of formoterol (0.5 mg/30 ml) had no inhibitory effect on the differential cell counts in BALF, the high concentration of formoterol (1 mg/30 ml) induced a significant decrease in the concentrations of both total cells and neutrophils in BALF while the macrophage concentration was not affected (Fig. 1A–C). Pretreatment with the high concentration of budesonide (0.5 mg/15 ml) caused a significant decrease in the concentrations of total cells, neutrophils and macrophages in BALF. However, only the concentration of neutrophils in BALF was reduced by the low concentration of budesonide (0.25 mg/15 ml) (Fig. 1A–C).

**Figure 2:**
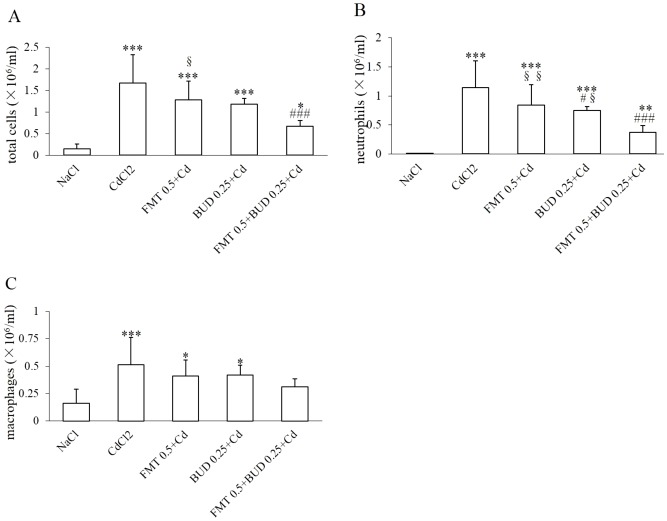
Effects of formoterol in combination with budesonide on the concentrations of total cells (A), neutrophils (B) and macrophages (C) in bronchoalveolar lavage fluid in rats exposed to cadmium. FMT 0.5+BUD 0.25+Cd: group pretreated with a combination of 0.5 mg/30 ml formoterol and 0.25 mg/15 ml budesonide before cadmium. For other abbreviation meaning: see Fig. 1 legend. * Indicates a significant difference in comparison with sham group (* P<0.05, ** P<0.01, *** P<0.001); # indicates a significant difference in comparison with cadmium-exposed group (# P<0.05, ## P<0.01, ###P<0.001); § indicates a significant difference in comparison with FMT 0.5+BUD 0.25+Cd group (§ P<0.05, §§ P<0.01).

A marked and very significant reduction of total inflammatory cell counts in BALF was observed when the low concentrations of formoterol and budesonide were combined. The inhibitory effect of the low concentration of budesonide on the number of neutrophils was significantly amplified when this drug was combined with the low inefficient concentration of formoterol (Fig. 2A–B). Though the combination did not induce any significant decrease in macrophage count compared to the animals exposed to cadmium, this parameter was no more significantly different when compared with the sham group (Fig. 2C).

### Effects of formoterol and/or budesonide on lung injuries evaluated by light microscopy and lung histomorphometry

**Figure 3:**
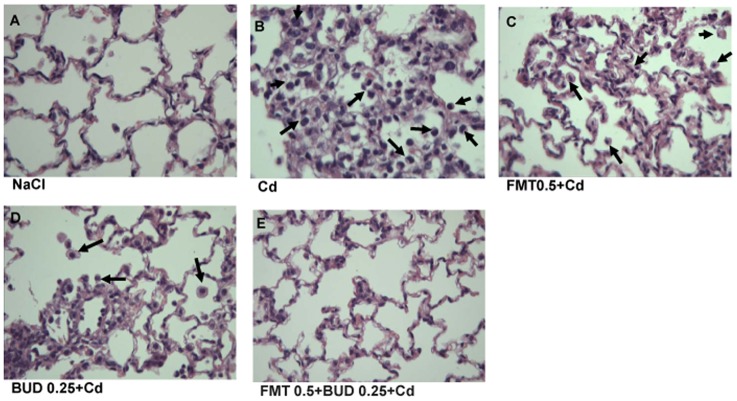
Effects of pretreatment with formoterol and/or budesonide on the histological injuries induced by cadmium inhalation. Representative histological sections of different groups are shown. A: sham group; B: cadmium-exposed group; C: FMT 0.5+Cd group; D: BUD 0.25+Cd group; E: FMT 0.5+BUD 0.25+Cd group. All sections are stained with hematoxylin–eosin and shown at ×200. Arrows indicate inflammatory cell infiltration into alveoli.

Concerning the histological examination, the lung architecture was preserved in sham group, with intact parenchyma and absence of inflammatory injury. Marked pathological changes in the peribronchiolar regions and throughout the alveoli, characterized by neutrophil and macrophage infiltration within alveoli and pulmonary parenchyma were observed. Focal congestion and hemorrhage were also sometimes observed in some portions of the cadmium-exposed lungs. Figure 3A and 3B shows representative lung tissue from rats exposed respectively to saline or cadmium.

**Figure 4:**
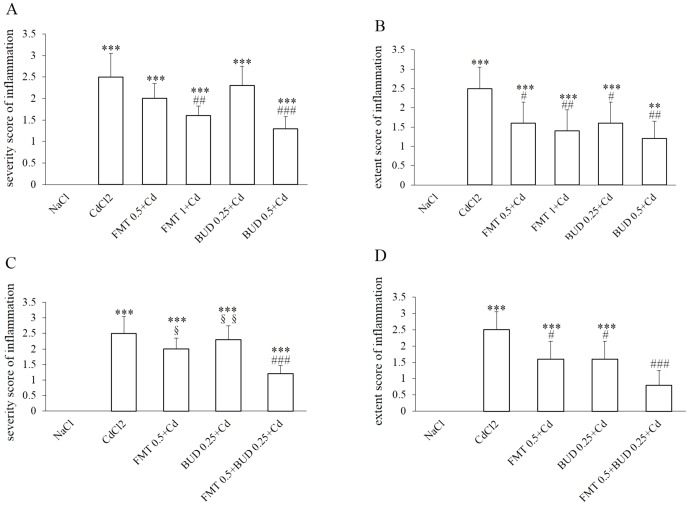
Inflammatory scores attributed to the severity (A, C) and extent (B, D) of histological injuries. For abbreviation meaning: see Fig. 1 and Fig. 2 legend. * Indicates a significant difference in comparison with sham group (* P<0.05, ** P<0.01, *** P<0.001); # indicates a significant difference in comparison with cadmium-exposed group (# P<0.05, ## P<0.01, ### P<0.001). § indicates a significant difference in comparison with FMT 0.5+BUD 0.25+Cd group (§ P<0.05, §§ P<0.01).

As demonstrated by the significant decrease in the scores attributed to the extent and severity of inflammatory cell infiltration, a significant reduction of histological lung injuries, especially neutrophil and macrophage infiltration within alveoli, were observed in rats pretreated with the high concentration of formoterol (Fig. 4A–B). However, only a reduced extent of inflammatory injuries was detected in rats pretreated with the low concentration of formoterol while the severity was not statistically affected by this pretreatment (Fig. 3C, Fig. 4A–B). Similar dose-dependent effects on lung injuries were observed in rats pretreated with different concentrations of budesonide. The low concentration of budesonide did not diminish the cadmium-induced lung injuries, except a decrease of the mean score attributed to the extent of inflammatory cell infiltration (Fig. 3D, Fig. 4A–B). As indicated by the inflammatory scores of severity and extent, the high concentration of budesonide significantly diminished the neutrophilic infiltration within the alveoli and in alveolar interstitium (Fig. 4A–B). The combination of both agents at low concentrations markedly protected the lungs against the inflammatory effects of cadmium (Fig. 3E). While both drugs were without effect on the severity of lung injuries when inhaled at low concentrations, this parameter was significantly reduced when animals were pretreated with the combination indicating a potentiated action on this key parameter (Fig. 4C). The extent of inflammation was no longer significantly different from that of the sham group (Fig. 4D).

### Effect of formoterol and/or budesonide on MMP-9 activity in BALF

**Figure 5:**
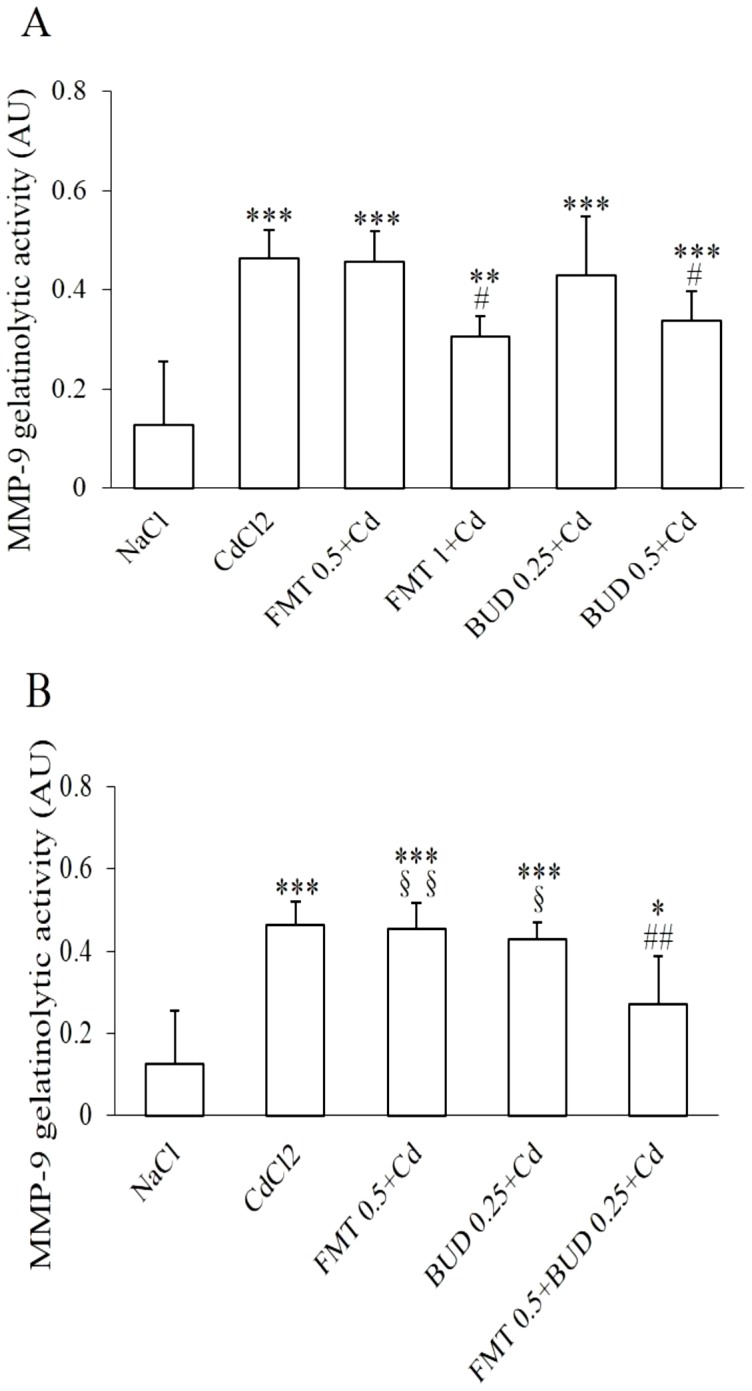
Effects of formoterol and/or budesonide on MMP-9 gelatinolytic activity in BALF. For abbreviation meaning: see Fig. 1 and Fig. 2 legend. * Indicates a significant difference in comparison with sham group (* P<0.05, ** P<0.01, *** P<0.001); # indicates a significant difference in comparison with cadmium-exposed group (# P<0.05, ## P<0.01). § indicates a significant difference in comparison with FMT 0.5+BUD 0.25+Cd group (§ P<0.05, §§ P<0.01).

A significant increase in MMP-9 gelatinolytic activity was detected in BALF after a single exposure of cadmium, while its activity was very low and even undectable in the sham group (Fig. 5A). Neither formoterol nor budesonide exerted any significant influence on MMP-9 activity in healthy rats (data not shown).

**Figure 6:**
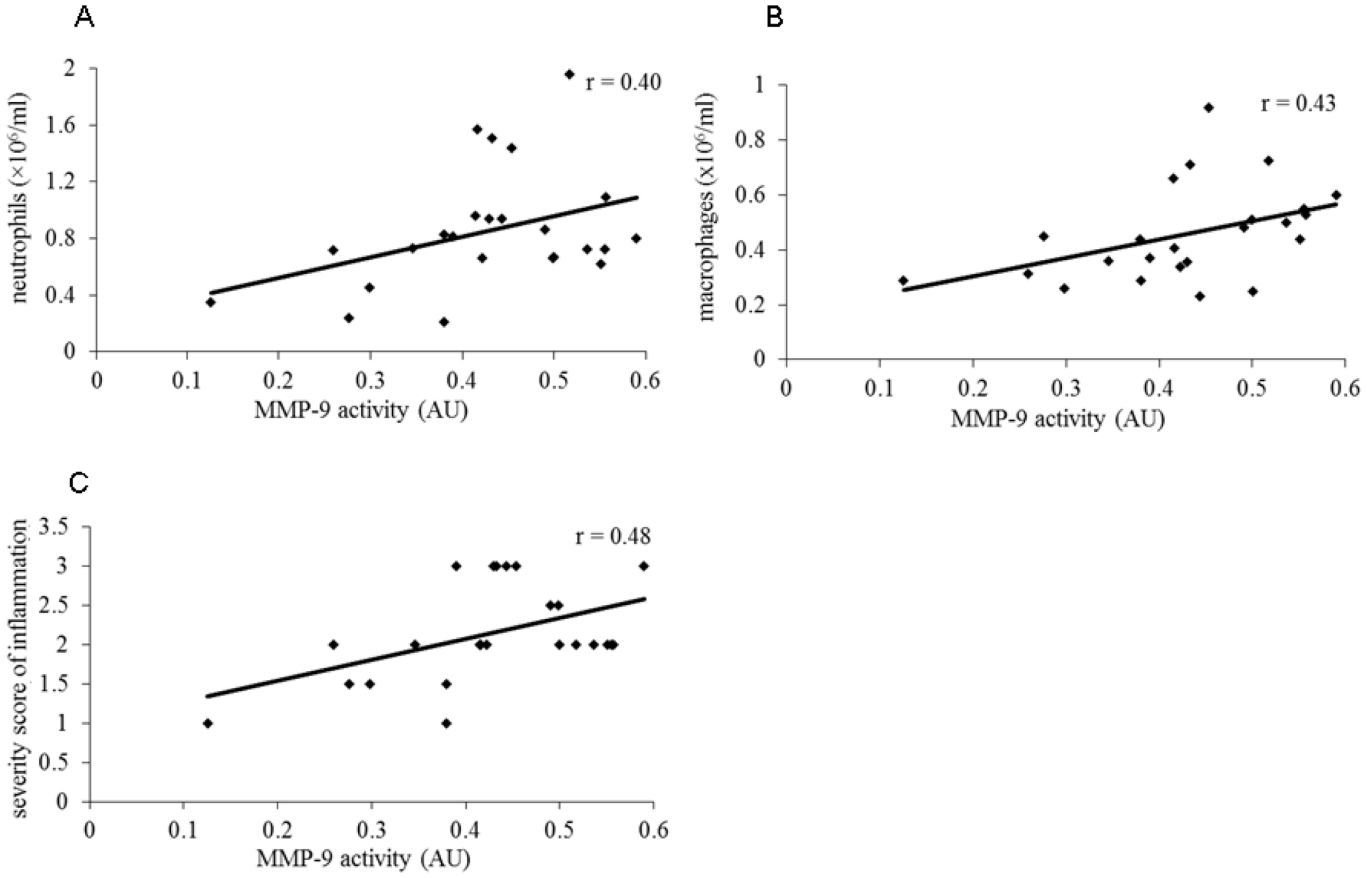
Linear correlation between MMP-9 activity with inflammatory cell counts and severity score of inflammatory injuries. The relationship between individual values of MMP-9 activity and the different inflammatory parameters obtained in cadmium group and in rats pretreated with low concentrations of formoterol and/or budesonide is represented. Coefficient of correlation is indicated for each figure.

As shown in figure 5A, pretreatment with the high concentrations of formoterol and budesonide resulted in a marked decrease in MMP-9 activity while the low concentrations of both drugs failed to prevent the cadmium-induced increase in MMP-9 activity. No significant correlation between MMP-9 activity and inflammatory markers measured in the present study was found in rats pretreated with formoterol. However, the modulation of MMP-9 activity was respectively correlated with neutrophil (r = 0.54, P<0.05) and macrophage concentrations (r = 0.65, P<0.01) in BALF when calculated with values obtained in cadmium group, and in rats pretreated with budesonide. Similar correlation was also detected for the severity of inflammatory infiltration (r = 0.50, P<0.05) when budesonide was administrated. A significant inhibitory effect on MMP-9 activity was observed in rats pretreated with the combination of both drugs (Fig. 5B). As illustrated in figure 6, this enhanced inhibitory effect on MMP-9 activity induced by combination of inefficient concentrations of both drugs was directly correlated with neutrophil (r = 0.40, P<0.05) and macrophage concentrations (r = 0.43, p<0.05) in BALF and severity score of inflammation (r = 0.48, P<0.05) when calculated with individual values obtained in cadmium group and in rats pretreated with the low concentrations of formoterol and budesonide alone and in combination (Fig. 6A–C).

### Effects of formoterol and/or budesonide on cytokine and chemokine levels in BALF

Cadmium exposure did not induce any significant variation in IL-1β, TNF-α and IL-8 in BALF as compared to sham group (data not shown).

## Discussion

While being very potent anti-inflammatory drugs, GCs are also known to be sometimes poorly active against neutrophilic pulmonary inflammation as that occurring in COPD patients especially during exacerbations [24]. The aim of this paper was to investigate whether their activity can be enhanced when associated with a LABA in rats exposed to a single inhalation of cadmium through a down-regulation of MMP-9 which has been shown to play a key role in this model [10].

Tobacco smoke being a main source of cadmium for non-occupationally exposed population, active smokers are thus highly exposed to this pollutant but passive smokers are also concerned as confirmed by high indoor air concentrations due to environmental tobacco smoke [2]. Occupationally exposed workers can also be detected in industries using cadmium in their manufacturing process such as nickel-cadmium battery production, electronic components, metallurgy and the majority of cadmium present in atmosphere is the result of fossil fuel combustion and municipal waste incineration [3]. Inhaled cadmium thus became a major risk for public health. Environmental exposure to cadmium and blood levels detected in exposed populations have been related with the worsening FEV1% predicted values [8]. Several evidences also suggest the involvement of cadmium in acute pulmonary inflammation but also in OLD including COPD associated with oxidative stress, chronic pulmonary inflammation and airspace enlargements [5]–[8]. Despite the lack of precise information about the particle size and concentration of cadmium inhaled by animals, acute exposure of rats to cadmium can be considered relevant to mimic some of the main features of the pathophysiological changes during the exacerbations of this disease and to investigate the influence of pharmacological agents.

Pulmonary deleterious effects induced by single or repeated cadmium inhalation have been related with up-regulation of various mediators and cytokines as GM-CSF, IL-6 [34], [35]. MMPs are also involved in air pollutants-induced pulmonary inflammation by acting on matrix remodeling and modulation of inflammation and cell signaling [36]. Within the MMP family, MMP-9 is suspected to play a predominant role in several murine models of acute lung injury but also in patients with acute lung inflammation [16], [37]–[39]. Elevated expression of MMP-9 has been observed in murine embryonic liver cells BNL CL2 stimulated by cadmium as well as in dogs exposed to cadmium inhalation leading to a marked neutrophilic inflammation [21], [40]. Consistent with these findings, MMP-9 has also been shown to be involved in the model used in this study as previously demontsrated by the anti-inflammatory effects provided by the inhibition of MMPs activity [10], [11]. Inflammatory cells including macrophages were demonstrated to be a major source of MMP-9 as well as resident cells [12], [13], [15], [41] explaining why our study was focused on this key enzyme showing an increased activity in response to acute exposure to cadmium.

The two pharmacological compounds selected for this study are very commonly used in human therapy as well the route of administration. Ideally, the pharmacological effects of inhaled drugs should be related to the drug concentrations in the lung tissue or to the drug intake to construct dose-response curves. However, inhalation of chemical compounds makes the exact determination of these parameters in small rodents very difficult [42]. The drug intake being unknown in our study, the amplitude of the effects was related to the concentrations of the nebulized solutions as performed in other studies [18], [20], [28], [42]. In a previous study, it was shown that the high concentration of formoterol (1 mg/30 ml) we used in the present work was able to partially protect the lungs against the bronchoconstrive effects of metacholine in healthy rats and to exert a protective effect against a single dose cadmium-induced acute pulmonary inflammation [11]. The lower concentration (0.5 mg/30 ml) of formoterol exerted a mild bronchodilatory effect with slight or no protective effect against cadmium-induced inflammation. Selection of these concentrations in our study aimed to investigate relevant pharmacological concentrations with likely no additional side effects. Budesonide is a very potent anti-inflammatory agent in rats as demonstrated by Birrell et al. [43] who reported a dose-related significant inhibitory effects of budesonide, ranging 0.3–10 mg/kg and administrated intratracheally, on the lipopolysaccharide (LPS)-induced lung tissue neutrophilia and on a broad range of inflammatory mediators such as MMP-9, TNF-α and IL-1β. An optimal inhibition was already obtained at doses as low as 1 mg/kg in this murine model of LPS-induced lung inflammation. During preliminary dose titration assays, a range of concentrations of budesonide has been evaluated. The two concentrations (0.25 mg/15 ml and 0.5 mg/15 ml) selected to exert a low to moderate effect in the present study were finally identical to those of formoterol, considered as a not very powerful anti-inflammatory agent. This likely reveals the low sensitivity of heavy metals-induced pulmonary inflammation to GCs suggested by previous studies in rats and dogs exposed to cadmium and to betamethsone and prednisolone respectively [20], [21].

The ability of GCs to modulate the protease/anti-protease balance in the lungs seems to markedly influence their efficacy and may vary depending on the causal factors of pulmonary inflammation. The suppression of elevated MMP-2 and MMP-9 levels by GCs associated with their anti-inflammatory action has been reported in both animal models of acute lung injury and in inflammatory cells stimulated respectively by LPS and H_2_O_2_ [22], [31], [44], [45]. By contrast, dexamethasone exerted no action on MMP-9 release by blood neutrophils stimulated with N-formylmethionyl-leucyl-phenyl-alanine (fMLP) [19] and fluticasone failed to decrease the release of MMP-9 and MMP-2 by human neutrophils stimulated with cigarette smoke medium known to lead to refractory inflammation in COPD patients [30]. In the same way, the inability of betamethasone to rebalance MMP-2-9/TIMP-1-2 ratio has been related to its inability to prevent the development of pulmonary emphysema associated to cadmium inhalation for 5 weeks [20] and a similar mechanism has been described in dogs [21].

It can thus be hypothesized (i) that a limited down-regulating effect on MMPs activity could thus contribute to limit the anti-inflammatory effects of GCs and (ii) that any drugs able to restore their action on MMPs activity would contribute to restore their anti-inflammatory effects. Our data provide additional convincing arguments in favour of this first hypothesis. Indeed, the protective effect against the cadmium-induced lung injuries only occured with the high concentration of budesonide able to inhibit the activity of MMP-9 and strong correlations between the inflammatory markers measured in the present study and MMP-9 activity calculated taking into account the budesonide groups were obtained. The fact that a mild anti-inflammatory effect can be induced by the low concentration of budesonide without significant influence on MMP-9 activity suggests that other mechanisms of action might be involved. The second hypothesis is supported by data provided by formoterol and budesonide combination. The pulmonary anti-inflammatory effects of β_2_-adrenoceptor agonists have been recently reviewed [25]. Briefly, these drugs can inhibit the inflammatory cell adhesion, the activation and the release of inflammatory mediators in various models of acute and chronic inflammation [46]. The influence of LABAs on pulmonary MMPs activity and expression related to pulmonary inflammation remains controversial. In contrast to the finding of Mortaz et al. [30] who found no influence of salmeterol on the release of elastase, MMP-2 and MMP-9 from human neutrophils stimulated with cigarette smoke, our previous studies provided evidences that inhibitory effects of formoterol on increased MMP-9 activity in BALF sampled in rats with acute and chronic pulmonary inflammation can be associated to a significant protective effect [11], [12]. The absence of correlation between MMP-9 activity and inflammatory markers in rats pretreated with formoterol in the present study can be explained by the more limited concentrations of formoterol than that tested previously ranging 1 mg/30 ml to 4 mg/30 ml. Taking into account these previous data, significant correlations between MMP-9 activity and neutrophil (r = 0.40, P<0.05) and macrophage (r = 0.5, P<0.01) concentrations in BALF as well as severity score of inflammatory injuries (r = 0.59, P<0.01) were detected. To check whether LABA can improve the protective effects of GCs in this relatively refractory experimental model, ineffective concentrations of budesonide and formoterol were combined before the cadmium inhalation. While the decrease in macrophage count in BALF was not significant, this combination exerted a significant and potentiated action on MMP-9 activity leading to a marked protective effect against pulmonary neutrophilic inflammation and lung injuries. The significant correlations between MMP-9 activity and inflammatory parameters calculated taking into account the animals treated with a combination of both drugs also argue in favor of this mechanism. The model used in the present study does not allow to determine whether the inhibited MMP-9 activity in BAL was the result of a direct action on producing cells or to an indirect action through a decrease in their number. As the major source of MMP-9 in this model being the macrophages which number remains unchanged, this last hypothesis seems unlikely.

In conclusion, high concentrations of inhaled formoterol or budesonide protect the lungs against the cadmium-induced acute neutrophilic inflammation by reducing the parenchyma inflammatory infiltration of neutrophils. The reduced MMP-9 activity recorded upon the action of both drugs could contribute to this protective effect. Due to the limited action of both drugs in this model, lower concentrations of both drugs exert no or few anti-inflammatory effects. This lack of protective effects has been related to the absence of action on MMP-9 activity. When ineffective concentrations of both compounds are combined, the interaction of both drugs provides an enhanced protection against the cadmium-induced lung injuries likely through a down regulation of MMP-9 activity. The finding of this present study showing the potentiation of interaction between both drugs at low inefficient concentrations could provide a new therapeutic strategy for restoring GC sensitivity so as to well control heavy metals-induced pulmonary inflammation with a less additional side effects.

## Supporting Information

Checklist S1 **ARRIVE Guidelines Checklist.**
(DOC)
